# Contrast-Induced Nephropathy in Renal Transplant Recipients: A Single Center Experience

**DOI:** 10.3389/fmed.2017.00064

**Published:** 2017-05-26

**Authors:** Bassam G. Abu Jawdeh, Anthony C. Leonard, Yuvraj Sharma, Swapna Katipally, Adele R. Shields, Rita R. Alloway, E. Steve Woodle, Charuhas V. Thakar

**Affiliations:** ^1^Division of Nephrology and Hypertension, Kidney C.A.R.E. Program, University of Cincinnati, Cincinnati, OH, United States; ^2^Cincinnati VA Medical Center, Cincinnati, OH, United States; ^3^Department of Family and Community Medicine, University of Cincinnati, Cincinnati, OH, United States; ^4^Division of Nephrology and Hypertension, Henry Ford Hospital, Detroit, MI, United States; ^5^Indiana University Health Ball Memorial Hospital, Muncie, IN, United States; ^6^Division of Transplant Surgery, The Christ Hospital, Cincinnati, OH, United States; ^7^Division of Transplant Surgery, University of Cincinnati, Cincinnati, OH, United States

**Keywords:** contrast nephropathy, transplant, AKI, calcineurin inhibitors, kidney

## Abstract

**Background:**

Contrast-induced nephropathy (CIN) in native kidneys is associated with a significant increase in mortality and morbidity. Data regarding CIN in renal allografts are limited, however. We retrospectively studied CIN in renal allografts at our institution: its incidence, risk factors, and effect on long-term outcomes including allograft loss and death.

**Methods:**

One hundred thirty-five renal transplant recipients undergoing 161 contrast-enhanced computed tomography (CT) scans or coronary angiograms (Cath) between years 2000 and 2014 were identified. Contrast agents were iso- or low osmolar. CIN was defined as a rise in serum creatinine (SCr) by >0.3 mg/dl or 25% from baseline within 4 days of contrast exposure. After excluding 85 contrast exposures where patients had no SCr within 4 days of contrast administration, 76 exposures (CT: *n* = 45; Cath: *n* = 31) in 50 eligible patients were analyzed. Risk factors assessed included demographics, comorbid conditions, type/volume of contrast agent used, IV fluids, *N*-acetylcysteine administration, and calcineurin inhibitor use. Bivariate and multivariable analyses were used to assess the risk of CIN.

**Results:**

Incidence of CIN was 13% following both, CT (6 out of 45) and Cath (4 out of 31). Significant bivariate predictors of CIN were IV fluid administration (*p* = 0.05), lower hemoglobin (*p* = 0.03), and lower albumin (*p* = 0.02). In a multivariable model, CIN was predicted by *N*-acetylcysteine (*p* = 0.03) and lower hemoglobin (*p* = 0.01). Calcineurin inhibitor use was not associated with CIN. At last follow-up, CIN did not affect allograft or patient survival.

**Conclusion:**

CIN is common in kidney transplant recipients, and there is room for quality improvement with regards to careful renal function monitoring post-contrast exposure. In our study, *N*-acetylcysteine exposure and lower hemoglobin were associated with CIN. Calcineurin inhibitor use was not associated with CIN. Our sample size is small, however, and larger prospective studies of CIN in renal allografts are needed.

## Introduction

Since contrast-induced nephropathy (CIN) was recognized more than 70 years ago, there have been continuous efforts to chemically modify iodine contrast media (CM) to become less nephrotoxic (1). Although CM have become safer, which reduces the likelihood of CIN per procedure, the indications for their use have dramatically increased. Over the last two decades, the utilization of computed tomography (CT) scanning and coronary angiograms (Cath) have increased by about 800 and 400%, respectively, with currently about 1.2 million Caths performed in the US annually and over 80 million CM doses delivered worldwide (2, 3). Furthermore, the number of patients with CIN risk factors, such as chronic renal insufficiency (CRI), diabetes, and congestive heart failure, has also grown. Currently, more than 27 million people are estimated to have CRI in the United States, and 200 million have diabetes worldwide (4, 5). The combination of increased CM administration frequency and greater prevalence of at-risk patients is likely to result in a continuing increase in CIN events. The incidence of CIN varies between studies, depending on risk factors of the cohort and definition of CIN, but figures have been reported to be as high as 50% in studies enriched with CRI and diabetic patients.

CIN is associated with a significant increase in mortality and morbidity in patients with native kidneys. After adjusting for comorbid factors, a retrospective study of 7,586 patients showed a significant increase in in-hospital and long-term mortality in patients who developed CIN (6). Another study of 1,826 patients, who underwent coronary artery intervention procedures, showed that 14% developed CIN and 1% required hemodialysis. Mortality was 1% in patients who did not develop CIN versus 7% in those with CIN, and 36% in the hemodialysis-treated CIN group (7). Moreover, studies by several other groups also support the position that CIN is associated with increased in-hospital and long-term mortality (8–10). Given the enhanced mortality and morbidity associated with CIN, several groups have been studying the pathophysiology of CIN, determining associated risk factors and investigating strategies for preventing it.

Although published papers studying CIN in native kidneys are abundant in the medical literature, data pertinent to CIN in renal allografts are relatively scarce (11–15). Moreover, it is plausible that renal transplant recipients is at a particularly higher risk for developing CIN due to aspects that are unique to renal allografts such as immunosuppression, lack of sympathetic innervation, glomerular hyperfiltration, and burden of cardiovascular disease. As a result, a better understanding of the incidence and predictors of CIN in renal transplant recipients is warranted.

In this retrospective study, we report the incidence of CIN in renal transplant recipients, its associated risk factors, and effect on long-term outcomes including allograft loss and death (16).

## Materials and Methods

This is a retrospective observational study that was approved by the University of Cincinnati (UC) Institutional Review Board in April 2013. All angiograms and other CM-enhanced studies performed on renal transplant recipients hospitalized between January 1st of 2000 and March 31st of 2014 were considered for the final analysis.

### Data Extraction, Entry, and Storage

After obtaining a list of all transplant recipients from the UC Medical Center prospective transplant database, subjects exposed to CM were identified by manually reviewing our institution’s electronic medical records including radiology and cardiac Cath lab records. After the list of CM recipients was completed, multiple variables including clinical data, laboratory results, and comorbid conditions were obtained and recorded in a password-protected Microsoft Excel format prior to converting to statistical output files. The choice of the variables was based on availability of the data, the prevalent knowledge related to risk factors of CIN, in addition to transplant-unique variables that might be associated with CIN (Table 1). The transplant- and immunosuppression-related data were imported from the transplant master database. The rest of the variables were obtained from the UC Medical Center electronic medical records. Since this was a retrospective record review, the study protocol did not influence the standard medical care received by the patients.

**Table 1:** **Baseline characteristics of patients with contrast media (CM) exposure**.

	All exposures (*N* = 76)	Contrast-induced nephropathy (CIN) (*N* = 10)	No CIN (*N* = 66)	*p*-Value
Gender (male)	50 (38)	30 (3)	53 (35)	0.18
Race (African-American)	16 (12)	30 (3)	14 (9)	0.31
Smoker	9 (7)	20 (2)	8 (5)	0.32
Diabetes	32 (24)	30 (3)	32 (21)	0.89
Hypertension	78 (59)	80 (8)	77 (51)	0.83
Congestive heart failure	7 (5)	10 (1)	6 (4)	0.69
Coronary artery disease	24 (18)	10 (1)	26 (17)	0.21
Hypotension (on day of contrast exposure)	8 (6)	20 (2)	6 (4)	0.30
ACE-I/ARB	17 (13)	10 (1)	18 (12)	0.47
Computed tomography versus Cath	59 (45)	60 (6)	59 (39)	0.95
CM groups				0.54
Low-osmolar CM	75 (57)	90 (9)	73 (48)	–
Isoosmolar CM	21 (16)	10 (1)	23 (15)	–
Non-ionic CM	4 (3)	0	5 (3)	–
IV fluid administration	53 (40)	80 (8)	48 (32)	0.048
*N*-acetylcysteine administration	36 (27)	60 (6)	32 (21)	0.09
Tacrolimus	70 (53)	80 (8)	68 (45)	0.36
Cyclosporine	13 (10)	20 (2)	12 (8)	0.40
Calcineurin inhibitor	83 (63)	100 (10)	80 (53)	0.20
Mycophenolate	97 (74)	100 (10)	97 (64)	1.00
Live donor kidney recipient	78 (59)	70 (7)	79 (52)	0.56
Allograft loss	9 (7)	10 (1)	9 (6)	0.93
Patient death	11 (8)	10 (1)	11 (7)	0.94
Age (years)	53.3 ± 15.4	49.6 ± 13.9	53.9 ± 15.6	0.41
Weight (kg)	82.0 ± 21.5	74.1 ± 31.3	83.2 ± 19.7	0.40
Height (")	68.1 ± 4.0	67.0 ± 4.5	68.2 ± 3.9	0.47
BMI (kg/m^2^)	27.2 ± 5.6	24.9 ± 6.8	27.5 ± 5.4	0.27
Baseline serum creatinine (SCr) (mg/dl)	1.47 ± 0.88	1.5 ± 0.92	1.5 ± 0.88	0.86
Post-contrast SCr peak (days 1–4) (mg/dl)	1.47 ± 0.85	2.1 ± 1.0	1.4 ± 0.79	0.055
SCr on most recent follow-up (mg/dl)	1.65 ± 1.2	1.9 ± 1.5	1.6 ± 1.2	0.61
Follow-up duration (months)	25.3 ± 24.7	33.4 ± 33.8	24.1 ± 23.1	0.91
Contrast volume (ml) (*N* = 72)	134 ± 69	122 ± 49 (*N* = 10)	136. ± 72 (*N* = 62)	0.45
Hemoglobin (g/dl)	10.3 ± 2.3	8.9 ± 2.1	10.5 ± 2.3	0.03
Albumin (g/dl) (*N* = 69)	3.5 ± 0.6	3.1 ± 0.49 (*N* = 9)	3.5 ± 0.62 (*N* = 60)	0.02
Graft age (days)	971 ± 1,103	1,013 ± 1,193	966 ± 1,099	0.78
Tacrolimus trough (ng/ml) (*N* = 47)	8.6 ± 5.2	10.5 ± 5.6 (*N* = 7)	8.3 ± 5.1 (*N* = 40)	0.37
Cyclosporine trough (ng/ml) (*N* = 10)	79 ± 19	89 ± 71 (*N* = 2)	76 ± 20 (*N* = 8)	0.16

*Total of 76 exposures in 50 patients. Numbers are % (count) or mean ± SD. Most significance tests are from Generalized Linear Models using Generalized Estimating Equations to account for multiple exposures in single patients. Tests for three CM groups, calcineurin inhibitors, and mycophenolate are Fisher’s exact tests not accounting for multiple exposures. Graft age and follow-up duration *p*-values are based on log-transformed raw values*.

### Definition of CIN

CIN was defined as a rise in serum creatinine (SCr) by >0.3 mg/dl or 25% from baseline within 4 days of CM exposure.

### Inclusion/Exclusion Criteria

All patients with functioning renal allografts and exposure to CM for cardiac or peripheral angiograms, contrasted CT scans, intravenous pyelograms, or any other CM-requiring intervention/study during their hospitalization at the UC Medical Center from January 1st of 2000 to March 31st of 2014 were eligible. Patients with no SCr available within 4 days post-CM exposure were excluded.

### Statistical Analysis

Descriptive statistics included means ± SDs for continuous variables and proportions and counts for categorical variables. All but three *p*-values were obtained from Generalized Linear Models employing Generalized Estimating Equations to account for clustering of multiple exposures within a patient. The remaining three tests (see Table 1) were performed where some cell frequencies were 0 or 100%—these were Fisher’s exact tests. *p*-Values associated with graft age and follow-up duration were based on logs of the raw values. A multivariable model with CIN as the dependent variable was constructed by starting with all relevant bivariate predictors associated with CIN at *p* < 0.20, then applying backward elimination of the weakest adjusted predictors until all remaining predictors had *p* < 0.05 in the presence of the other remaining predictors. The study alpha was a two-tailed *p* = 0.05, unadjusted for multiple tests, and all analyses were conducted using SAS 9.4.

## Results

Out of 161 CM exposures, 85 were excluded for not meeting inclusion/exclusion criteria. Fifty patients with 76 exposures (45 CT and 31 Cath) were analyzed. For 73 exposures, baseline SCr was established less than 4 months prior to exposure, while three baseline SCrs were established more than 4 months prior. Counting exposures (rather than patients), 50% were in male patients, 16% were in African-American patients, 9% were in smokers, 32% had diabetes, 78% had hypertension, 24% had coronary artery disease, 7% congestive heart failure, and 17% were on an ACE inhibitor. Seventy eight percent of exposures were in patients received living donor kidneys. Maintenance immunosuppression included tacrolimus in 70%, cyclosporine in 13%, and mycophenolate in 97% of the exposures (Table 1).

**Figure 1:**
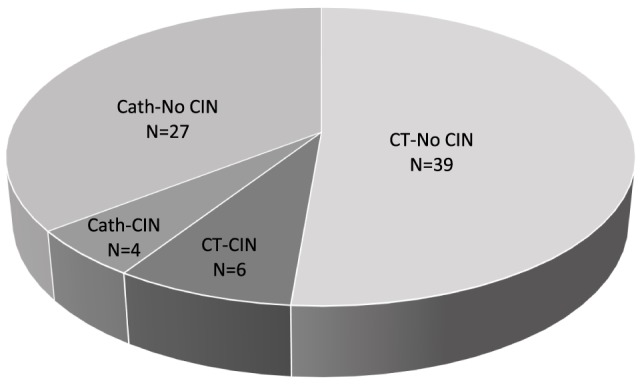
**Contrast-induced nephropathy (CIN) frequency**. Total number (*N*) of contrast media (CM) exposures = 76, *N* of CM exposures *via* computed tomography (CT) = 45, *N* of CM exposures *via* coronary angiogram (Cath) = 31.

At the time of CM exposure, the (mean, ±SD) age of the patients was 53 ± 15 years, baseline SCr, 1.5 ± 0.9 mg/dl, hemoglobin 10.3 ± 2.3 g/dl, and albumin 3.5 ± 0.6 g/dl. Tacrolimus 12-h trough was 8.6 ± 5.2 ng/ml, cyclosporine 12-h trough was 79.1 ± 19.2 ng/ml, and 8% of the patients were hypotensive (SBP < 100 mm Hg). IV fluids and *N*-acetylcysteine were administered in 53 and 36% of the patients, respectively (Table 1). The overall incidence of CIN was 13% (10 out of 76 exposures), equally so in patients who underwent Cath (4 out of 31 exposures) and in patients who received CM-enhanced CT scans (6 out of 45 exposures) (Figure 1).

Significant simple bivariate predictors of CIN were IV fluid administration (*p* = 0.05), lower hemoglobin (*p* = 0.03), and lower albumin (*p* = 0.02). Female gender (*p* = 0.18) and *N*-acetylcysteine administration (*p* = 0.09) also made the cutoff to be included as candidate predictors in a multivariable model. After these five predictors were entered into a multivariable prediction model and subjected to backwards selection, lower hemoglobin (*p* = 0.01) and *N*-acetylcysteine (*p* = 0.03) were associated with CIN (Table 2).

**Table 2:** **Predictors of contrast-induced nephropathy (CIN)**.

	Odds ratio (confidence interval)	*p*-Value
*N*-acetylcysteine administration	9.0 (2.7–29.9)	0.03
Hemoglobin per unit (g/dl)	0.55 (0.32–0.93)	0.01

*Significant predictors of CIN (*p* < 0.05) after a multivariable model with CIN as the dependent variable*.

Calcineurin inhibitor use was not associated with CIN. CIN was not associated with allograft loss or death on last follow-up, with last follow-up occurring (mean ± SD) 25 ± 24 months after exposure.

## Discussion

It is plausible that renal transplant recipients are at a particularly high risk for developing CIN. In addition to all the traditional risk factors associated with CIN in native kidneys, the following aspects are unique to renal allografts:

- Immunosuppressive regimens often include calcineurin inhibitors, which harbor a significant risk of nephrotoxicity. Calcineurin inhibitor nephrotoxicity is mediated by several mechanisms including afferent arteriolar vasoconstriction, peripheral arteriolar hyalinosis, isometric vacuolization of tubular epithelial cells, stripped interstitial fibrosis, and thrombotic microangiopathy, all of which might predispose to CIN (17).
- Mycophenolate mofetil (MMF)/mycophenolate sodium (MPA), an inosine monophosphate dehydrogenase inhibitor, is another medication that together with calcineurin inhibitors forms the backbone of modern maintenance immunosuppression regimens. MMF/MPA has been shown to ameliorate the production of reactive oxygen species (ROS) in hepatic ischemia reperfusion injury in Wistar rats (18). Moreover, mycophenolate has been shown to neutralize the calcineurin inhibitor effect in enhancing phenylephrine-induced vasoconstriction of abdominal aorta allografts in Lewis rats. This has been mediated by an increase in the endothelial nitric oxide (NO) synthase activity (19).
  CIN is mediated by an interplay of various factors that increase vasoconstriction and mitigate vasodilatation, leading to renal medullary hypoxia and acute tubular necrosis. Furthermore, CM administration accentuates the production of ROS. ROS then cause direct damage of the tubular epithelial cells and scavenge NO leading to further arteriolar vasoconstriction (20, 21). Therefore, based on our current understanding of the pathophysiology of CIN and the preclinical data demonstrating a negative effect of MMF/MPA on ROS, we speculate that MMF/MPA might have a protective role in CIN.
- Renal allografts lack sympathetic innervation that is normally responsible for a significant portion of sodium and water retention in the proximal tubules. As a result, transplant recipients might be prone to develop hemodynamic-mediated acute allograft insufficiency secondary to a decrease in the effective arterial circulating volume. Intrarenal hemodynamic changes in kidney allografts have been evaluated. A study compared resistive indices (RI), measured by Doppler studies, of donor kidneys with their subsequent RI in recipients. RI, used as a surrogate for intrarenal vascular impedance, improved after transplantation reflecting an increase in renal blood flow to maintain GFR (22). The authors explain their finding by the lack of sympathetic innervation in renal allografts. Given the effect of the sympathetic nervous system on systemic and renal hemodynamics, denervated allografts might have a different CIN susceptibility profile when compared to native kidneys.
- Renal allografts hyperfiltrate, undergo hemodynamic stress, and develop maladaptive structural changes. Therefore, despite maintaining near normal GFR by virtue of maximizing various compensatory mechanisms, renal allografts often have significantly reduced renal reserve. This makes them more prone for renal insults including CIN.
- In addition to the burden of cardiovascular disease leading to frequent coronary and peripheral angiograms, renal transplant recipients are immunosuppressed and at a higher risk for developing infections and malignancies, diagnosis of which often requires CM-enhanced studies.

Although CIN in native kidneys has been studied extensively, only few small retrospective studies addressed CIN in renal allografts (11–15). In a retrospective study of 35 renal transplant recipients on cyclosporine, the incidence of CIN defined by a rise in SCr by >25% was 21% (12). This is higher than the 13% CIN incidence seen in native kidneys; however, six out of the seven patients developing CIN received 1/2 normal saline or no IV fluids for prophylaxis as opposed to standard of care isotonic fluid administration (23). In another paper of 57 patients undergoing coronary angiograms, CIN defined as a rise in SCr by >25% or 0.5 mg/dl within 3 days post-Cath occurred in 16% of the patients (11). Univariate comparisons showed that the use of *N*-acetylcysteine and isoosmolar CM were protective. Logistic regression analysis, however, revealed that only low osmolarity CM administration was associated with CIN compared to isoosmolar CM (odds ratio of ~7.7). Type of renal graft, preexisting comorbidities, and immunosuppressive medications were not associated with CIN. Neither of the two mentioned studies looked at hard outcomes such as death-censored allograft loss or death. More recently, three small studies reported a CIN incidence of 6–13% (13–15). In one study, CIN occurred in 13% of renal transplant recipients undergoing endovascular aortic aneurysm repair versus 5% in a non-transplanted comparison group (14).

In our study of transplant recipients receiving CT or Cath, the incidence of CIN was 13%, which is consistent with what was published and reproduced before in both native and transplant kidneys (11, 14, 23). Anemia was independently associated with CIN, which is consistent with previous reports (24). This is biologically plausible since anemia can exacerbate hemodynamic stress and ischemia reperfusion-related acute kidney injury particularly in solitary allografts whose hemodynamic compensatory mechanisms are already exhausted. *N*-acetylcysteine use positively correlated with CIN, which contradicts multiple previous studies that showed it had a protective effect (25, 26). This protective effect of *N*-acetylcysteine, however, could not be reproduced in multiple subsequent trials including a very well-designed randomized controlled trial. In this prospective trial of 2,308 patients with risk factors for CIN, *N*-acetylcysteine failed to have a protective effect (23). An alternative explanation for our finding could be that *N*-acetylcysteine was given to patients who were considered by the treating physician as high risk for developing CIN. Therefore, we should be cautious inferring that *N*-acetylcysteine use is a predictive marker of CIN given the possibility of indication bias that exists in retrospective analyses. We anticipated that intravenous fluid administration will have a protective effect, which would persist after multivariable modeling, but this did not occur. We attribute this to the small sample size and potential underlying bias that could not be accounted for in a retrospective design.

We did not observe any safety signal related to calcineurin inhibitor use, and we could not assess any potential protective effect of mycophenolate since 97% of patients were on it.

We acknowledge that our study has several limitations. First, it is a small and retrospective study. Second, we did not capture CM-enhanced studies done as outpatient procedures or at outside hospitals. Third, we did not exclude acute kidney injury episodes that occurred early after transplantation. This could overestimate the incidence of CIN since early acute allograft failure could be confounded by several factors including rejection.

In conclusion, CIN is common in renal transplant recipients receiving both intra-arterial and intravenous CM. Therefore, there is room for careful risk stratification, optimizing hemodynamics and avoiding further potential nephrotoxins in transplant recipients receiving CM-enhanced studies. Prospective controlled studies of CIN in transplant settings are warranted.

## Author Contributions

BA oversaw study design and conduction and wrote the manuscript. AL performed statistical analysis and wrote the statistics section of the manuscript. YS, SK, and AS performed data collection and helped with statistical analysis and writing of the manuscript. RA and EW helped with study design, access to transplant master database, and writing the manuscript. CT helped with study design and critical review of the manuscript.

## Conflict of Interest Statement

The authors declare that the research was conducted in the absence of any commercial or financial relationships that could be construed as a potential conflict of interest.
